# *In silico* Drug Screening Approach Using L1000-Based Connectivity Map and Its Application to COVID-19

**DOI:** 10.3389/fcvm.2022.842641

**Published:** 2022-03-24

**Authors:** Takaharu Asano, Sarvesh Chelvanambi, Julius L. Decano, Mary C. Whelan, Elena Aikawa, Masanori Aikawa

**Affiliations:** ^1^Center for Interdisciplinary Cardiovascular Sciences, Cardiovascular Division, Department of Medicine, Brigham and Women's Hospital and Harvard Medical School, Boston, MA, United States; ^2^Center for Excellence in Vascular Biology, Cardiovascular Division, Department of Medicine, Brigham and Women's Hospital and Harvard Medical School, Boston, MA, United States; ^3^Department of Human Pathology, I.M. Sechenov First Moscow State Medical University of the Ministry of Health, Moscow, Russia; ^4^Channing Division of Network Medicine, Department of Medicine, Brigham and Women's Hospital and Harvard Medical School, Boston, MA, United States

**Keywords:** L1000, connectivity map (CMap), ACE2, COVID-19, drug repurposing, lung epithelial cell

## Abstract

Conventional drug screening methods search for a limited number of small molecules that directly interact with the target protein. This process can be slow, cumbersome and has driven the need for developing new drug screening approaches to counter rapidly emerging diseases such as COVID-19. We propose a pipeline for drug repurposing combining *in silico* drug candidate identification followed by *in vitro* characterization of these candidates. We first identified a gene target of interest, the entry receptor for the SARS-CoV-2 virus, angiotensin converting enzyme 2 (ACE2). Next, we employed a gene expression profile database, L1000-based Connectivity Map to query gene expression patterns in lung epithelial cells, which act as the primary site of SARS-CoV-2 infection. Using gene expression profiles from 5 different lung epithelial cell lines, we computationally identified 17 small molecules that were predicted to decrease ACE2 expression. We further performed a streamlined validation in the normal human epithelial cell line BEAS-2B to demonstrate that these compounds can indeed decrease ACE2 surface expression and to profile cell health and viability upon drug treatment. This proposed pipeline combining *in silico* drug compound identification and *in vitro* expression and viability characterization in relevant cell types can aid in the repurposing of FDA-approved drugs to combat rapidly emerging diseases.

## Introduction

Coronavirus disease 2019 (COVID-19), which is caused by the infection of severe acute respiratory syndrome coronavirus 2 (SARS-CoV-2), broke out in December 2019. The World Health Organization designated COVID-19 as a global pandemic in March of 2020. Since then, multiple variants have emerged, spread globally, and continue to hit the health, life, and economy of people worldwide even with the advent of various vaccines designed to provide immunity against the virus. Intensive research efforts have revealed that morbidity, severity, and mortality from COVID-19 are strongly associated with various cardiovascular comorbidities (1). Since COVID-19 infection of lung epithelium could directly signal toward an increased risk for cardiovascular diseases (2), reducing productive infection of these cells becomes an important strategy to not only counter acute infection but prevent progression to cardiovascular diseases. Acute infections of viruses such as influenza virus (3), HIV (4), and SARS-CoV-2 (5) have been shown to have a direct impact on cardiovascular health and act as the initial insult that increases the incidence of cardiovascular disease in these patients. However, due to the massive number of patients globally infected by the virus, increase in cardiovascular disease prevalence due to infection with SARS-CoV-2 virus could severely burden the cardiovascular healthcare system in the future. Therefore, repurposing FDA approved drugs to contain SARS-CoV-2 infection can be an effective strategy to limit the risk of developing cardiovascular diseases in these patients.

The first step of SARS-CoV-2 invasion into human host cells is implemented by the SARS-CoV-2 spike protein binding to a host cell receptor, angiotensin-converting enzyme 2 (ACE2) (6). Inhibiting the spike protein and ACE2 interaction is, therefore, one of the promising drug targets for combating COVID-19 (7). Most current studies aim to inhibit the interaction by drugs that binds to the spike protein or ACE2 protein (7, 8).

In case drug targets are known, target-based drug discovery, in which a specific drug target that associates a target disease is identified and then hit compounds that interact with the target are searched for, is a proven strategy to generate new drugs (9). Conventional drug screening methods used in this strategy such as high-throughput screening (HTS), however, search for a limited number of small molecules that directly interact with the target protein. Moreover, this process can be slow, cumbersome and has driven the need for developing new drug screening approaches to counter rapidly emerging diseases like COVID-19.

The Connectivity Map (CMap) is a database of gene expression profiles induced by exposing a variety of cell types to various perturbagens including small molecules and has been expanded to have over one million gene expression profiles using over 20,000 small molecules through the introduction of L1000 assay technology (10, 11). L1000-based CMap has been widely used for rapid drug repurposing and the core idea is to identify small molecules that induce a gene expression profile canceling or mimicking the differential gene expression caused by diseases (12, 13). This approach is a kind of phenotypic screening, which is a counter approach to the target-based drug discovery and identifies small molecules that provide nice phenotypes (e.g., gene expression) to cells or animals first and then investigates the mechanism. Phenotypic screening has attracted attention recently because it was shown to be the most successful approach for first-in-class drugs (9, 14). As described above, although the conventional L1000-based CMap approach is an attractive way to find drugs that the conventional methods could overlook, it hardly has been applied to target-based drug discovery because this approach requires decreased and/or increased gene set, not a single target gene.

In this study, we propose a pipeline for drug repurposing that applies the L1000-based CMap to a single gene target, ACE2 which is the entry receptor for the SARS-CoV-2 virus. Using gene expression profiles from 5 different lung epithelial cell lines which act as the primary site of SARS-CoV-2 infection, we computationally identify small molecules that were predicted to decrease ACE2 expression. We further perform a streamlined validation in the normal human epithelial cell line BEAS-2B to identify the potential of these compounds to decrease ACE2 surface expression as well as profile cell health and viability upon drug treatment. This proposed pipeline combining *in silico* drug compound identification and *in vitro* expression and viability characterization in relevant cell types can aid in the repurposing of FDA-approved drugs to combat rapidly emerging diseases.

## Materials and Methods

### L1000-Based CMap Dataset

Level 5 gene expression profiles of L1000-based CMap were downloaded from GSE92742 and GSE70138. This dataset has the gene expression profiles in a total of 591,697 conditions consisting of various combinations of perturbagens, cell types, doses, and time points. The profile values mean mRNA expression levels compared to control (the background of the plate). Each gene expression profile comprises 12,328 genes, 978 of which are measured directly (called landmark genes). Of the remaining genes, 9,196 are well-inferred genes the expression levels of which correlate to the actual measured levels with *p*-values ≤0.05, and the other 2,154 less-well inferred genes. ACE2 is in the well-inferred genes.

### Cell Culture and Reagents

BEAS-2B normal human epithelial cell line was purchased from ATCC (Catalog number: CRL-9609) and cultured according to vendor instructions using BEGM kit from LONZA (Catalog number: CC-3170). Cells were cultured on 96-well black μ-plate from ibidi (Catalog Number: 89626) for imaging studies. Tanespimycin (abcam ab141433), Acetylcysteine (Cayman, 20261), Amifostine (Cayman 14398), Bortezomib (Ayman 10008822), FK-866 (Cayman 13287), Gemcitabine (Cayman 11690), Idarubicin (Cayman 14176), NVP-AUY922 (Cayman 10012698), NVP-BEZ235 (Cayman 10565), PIK-75 (Cayman 10009210), SN-38 (Cayman 15362), Tretinoin (Cayman 11017), YM-155 (Cayman 11490), Ingenol (Cayman 14031), Sulforaphane (LKT S8044), CD-437 (Sigma C5865), and Parbendazole (Sigma 1498706) were dissolved in DMSO. 1000x concentration working solution was used for downstream experimentation.

### Immuno-Fluorescent Staining With High Content Imaging (HCI) for Quantifying ACE2 Expression

BEAS-2B cells were treated overnight with indicated drugs at indicated doses. Cells were then washed twice with PBS and fixed in 4% Paraformaldehyde for 15 min at room temperature. Cells were then stained with primary anti-human ACE2 antibody (Abcam ab239924) or isotype control for 1 h at 4°C with gentle shaking. Cells were then washed thrice with PBS and stained using AF555 labeled secondary antibody. Hoescht 33342 was used as nuclear counterstain.

Sixteen images were captured per well using 20x objective of an Image Express Pico (Molecular Devices) and analyzed using 2 color cell scoring system. Isotype control stained well was used to identify threshold for detecting ACE2 positivity in cells.

### Cell Viability Measurement

BEAS-2B cells were plated in 96 well plates at 70% confluency and treated for 48 h with each drug at indicated doses. Cell mitochondrial activity was profiled using CyQuant MTT Cell Viability Assay (Thermo Fisher, Catalog Number V13154) following manufacturer instructions. The absorbance at 590 nm was quantified using Spectramax i3 (Molecular Devices). Cytotoxicity was quantified using CyQuant LDH Cytotoxicity Assay (Thermo Fisher Catalog Number C20301) following manufacturer instructions. Briefly, 50 μl of media supernatant from each well was used to quantify cell toxicity and was normalized to cells lysed with 10x cell lysis buffer as 100% cell death. Absorbance was measured at 490 nm and 680 nm with the 680 nm absorbance used to determine background plate absorbance. Mitochondrial Super Oxide production was quantified using MitoSOX™ Red Mitochondrial Superoxide Indicator (Thermo Fisher Catalog Number M36008) using manufacturer instructions. Hoescht 33342 was used as a nuclear counterstain. Sixteen images were captured per well using 20x objective of the Image Express Pico and analyzed using 2 color cell scoring system to determine average Mitochondrial Superoxide Intensity per cell.

### FACS Staining

BEAS-2B cells were treated with indicated doses of drugs overnight and cells detached using accutase. Cells were resuspended in Stain Buffer with FBS (BD Biosciences) and stained with Fixed Viability Stain (FVS-780 BD Biosciences) followed by staining with ACE2-AF647 antibody (Biolegend). Cells stained with isotype AF647 antibody (BD Biosciences) were used to draw gates for ACE2 positivity. Cells were acquired on Cytek Aurora and data analyzed using Flowjo 10.8.

### Statistical Methods

ACE2 expression and cell viability data were analyzed using a Python library, SciPy. FACS data was analyzed using Graphpad Prism 9.0. Student's *t*-test with Welch's correction was used to compare the effect of each treatment to control (vehicle) treated samples. Comparisons between the effects of each candidates were not performed.

## Results

### Preprocessing for Drug Screening

**Figure 1:**
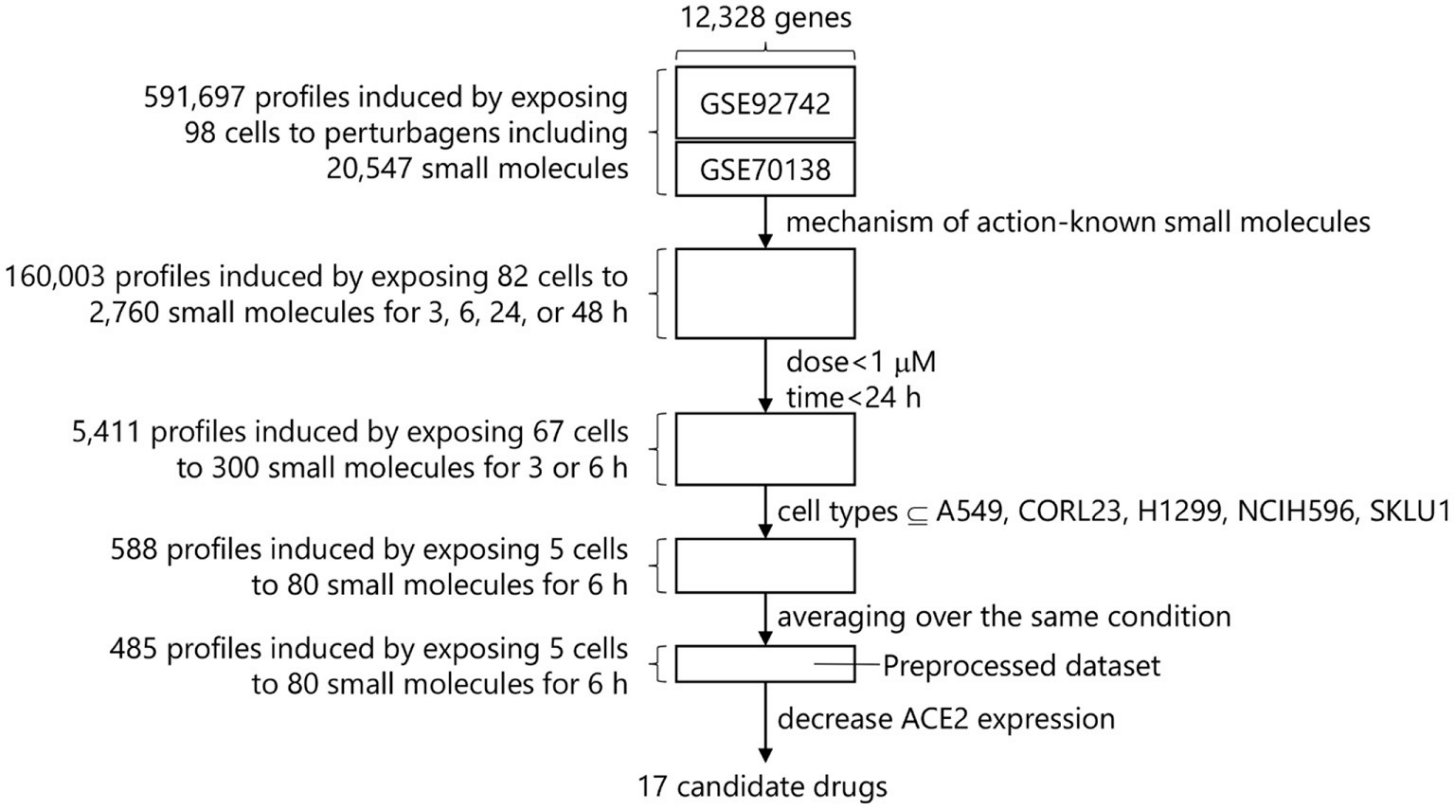
Drug screening process in L1000-based CMap.

The following filters were applied to the L1000-based CMap dataset to identify small molecules that are effective to COVID-19 therapy before searching for small molecules that decrease ACE2 expression (Figure 1).

#### Perturbagen Selection

A perturbagen is a reagent used to treat cells and measure the resulting biological response includes CRISPR/Cas9 constructs, short hairpin RNA (shRNA), open reading frames (ORFs), biological agents, small molecules, and so forth. The goal of this study was to identify small molecules suitable for rapid drug repurposing. Drug Repurposing Hub is a curated and annotated collection of FDA-approved drugs, clinical trial drugs, and pre-clinical tool compounds with a companion information resource, and the mechanisms of action of 6,232 drugs are explicitly stated in it (drug information version: 3/24/2020) (15). L1000-based CMap dataset contains 20,547 small molecules, and 2,760 small molecules out of them overlap with these 6,232 drugs. We therefore extracted the gene expression profiles in conditions treated with these 2,760 small molecules, resulting 160,003 profiles induced by exposing 82 cell types to 2,760 small molecules with up to 177.6 μM for 3, 6, 24, or 48 h.

#### Dose Selection

Each small molecule was measured at various dosages within the database. To compare these dosages and to increase ease of handling, doses from a similar range were converted to a single value as indicated in Supplementary Table 1. Among available dosages, 0.0001–100 μM, the most used dosage was 10 μM and the profiles using over 1 μM dosage dominate 68.5% of the total. These dosages over 1 μM were eliminated due to concerns about cytotoxicity and the need to identify drugs that would work at low doses and minimal side-effects to reduce gene expression of ACE2.

#### Time-Point Selection

Drugs for COVID-19 need to work within hours to reduce ACE2 expression since that would be the ideal window for drug intervention of a patient testing positive for SARS-CoV-2. The available time-points were 3, 6, and 24 h after the dose selection. We removed the timepoints over 24 h to identify fast-acting drug candidates. This step provided 5,411 profiles induced by exposing 67 cell types to 300 small molecules with 0.0003–0.3162 μM for 3 or 6 h.

#### Cell Type Selection

After the time-point selection, 67 cell types derived from 14 organs such as large intestine, lung, breast, etc. were available. Each cell type shows a different gene expression profile even though the same small molecule is applied. It is advisable to use the gene expression profiles in a specific cell type of interest. However, human lung epithelial cells such as BEAS-2B that are suitable for model host cells in COVID-19 study are not included in L1000-based CMap. We thus selected 5 cell types (A549, CORL23, H1299, NCIH596, and SKLU1) derived from lung in the dataset. Of note, all five cell types were from lung epithelial cell lines derived from various tumors. The gene expression profiles in conditions using these 5 cell types were extracted, resulting 588 profiles induced by exposing 5 cell types to 80 small molecules with 0.0003–0.3162 μM for 6 h.

The 588 profiles came from 485 unique conditions. Finally, the gene expression levels were averaged over the same conditions, resulting the preprocessed dataset that has 485 profiles induced by exposing 5 cell types to 80 small molecules with 0.0003–0.3162 μM for 6 h.

### Identification of Small Molecules That Decrease ACE2 Expression

We focused on the expression levels of ACE2 and extracted the combinations of the small molecules and their dosages that decrease ACE2 expression (i.e., show the negative ACE2 expression levels) in each cell type. As for A549, since 214 combinations were identified, the top 10 combinations with the lower ACE2 expression levels were selected. In the other 4 cell types, 4, 4, 6, and 5 combinations were identified in CORL23, H1299, NCIH596, and SKLU1, respectively. Out of these 29, 19 combinations were unique. We removed 0.0032 μM veliparib which was commercially unavailable and 0.01 μM idarubicin, while keeping the larger dose, 0.1 μM idarubicin. As a result, 17 small molecules and their optimal dosages in 6 h were obtained as the drug repurposing candidates. The ACE2 expression levels in each cell type treated with these 17 small molecules with their optimal dosages are shown in Table 1.

**Table 1:** The identified 17 small molecules, their optimal doses, mechanisms of action, and ACE2 expression levels in each cell type.

**Drug name (uM)**	**Mechanism of action**	**Cell type**				
		**A549**	**CORL23**	**H1299**	**NCIH596**	**SKLU1**
Acetylcysteine (0.01)	Mucolytic agent	−2.29				
CD-437 (0.1)	Retinoid receptor agonist	−1.74				
NVP-BEZ235 (0.0316)	mTOR inhibitor, PI3K inhibitor	−1.47				
Amifostine (0.3162)	Reducing agent	−1.47				
Ingenol (0.01)	PKC activator	−1.45		No data		
NVP-AUY922 (0.1)	HSP inhibitor	−1.41				
Tretinoin (0.3162)	Retinoid receptor agonist, retinoid receptor ligand	−1.40				
Sulforaphane (0.001)	Anticancer agent, aryl hydrocarbon receptor antagonist	−1.32				
Bortezomib (0.0316)	NFkB pathway inhibitor, proteasome inhibitor	−0.54	0.13	0.13	−0.80	−0.96
Parbendazole (0.3162)	Tubulin polymerization inhibitor	−0.53	0.01	0.80	−0.80	−0.09
Idarubicin (0.1)	Topoisomerase inhibitor	−0.47	0.70	−1.44	0.00	0.46
Tanespimycin (0.3162)	HSP inhibitor	−0.23	0.00	0.09	0.39	0.07
SN-38 (0.3162)	Topoisomerase inhibitor	−0.19	−0.37	−0.55	−0.38	0.20
FK-866 (0.1)	Niacinamide phosphoribosyltransferase inhibitor	0.06	−0.79	0.41	−0.50	0.10
Gemcitabine (0.1)	Ribonucleotide reductase inhibitor	0.09	0.63	0.94	0.50	−1.28
YM-155 (0.3162)	Survivin inhibitor	0.36	0.00	−0.82	−0.57	−0.17
PIK-75 (0.1)	DNA protein kinase inhibitor, PI3K inhibitor	2.32	0.45	−1.04	0.54	−0.37

*The small molecules are sorted based on the ACE2 expression levels in A549. The numbers in brackets beside drug names are their optimal dosages (μM). Gray area means no data in the preprocessed dataset*.

NVP-AUY922 and tanespimycin are heat shock protein (HSP) inhibitors. NVP-BEZ235 and PIK-75 are PI3K inhibitors. CD-437 and tretinoin are retinoid receptor agonists. SN-38 and idarubicin are topoisomerase inhibitors. The other 9 small molecules have different mechanisms of action. The candidates cover a wide variety of mechanisms of action. On the other hand, most small molecules have been developed for cancer drugs except for acetylcysteine, ingenol, and sulforaphane.

For 9 among 17 small molecules, ACE2 expression levels were available in all 5 cell types (Table 1). The ACE2 expression levels were quite different in each cell even though the same small molecules are applied with the same doses, suggesting the other 8 small molecules whose ACE2 levels were available only in A549 also have different ACE2 expression levels depending on the cell types. On the other hand, these small molecules show negative ACE2 expression levels in at least 1 cell type, suggesting that these small molecules have a potential to decrease ACE2 expression in human lung epithelial cells.

**Figure 2:**
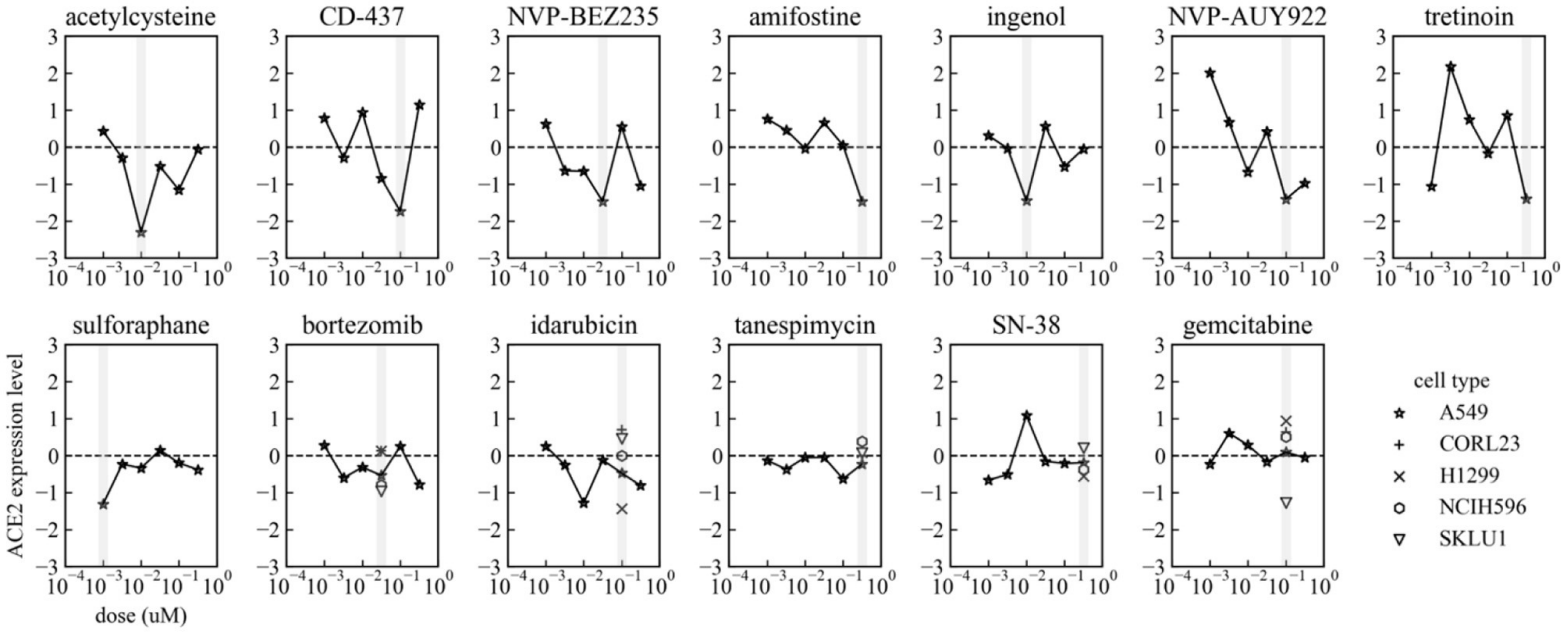
Dose-response of ACE2 expression levels in each small molecule in A549. Gray vertical bars mean the optimal dosages in each small molecule indicated in Table 1. ACE2 expression levels in CORL23, H1299, NCIH596, and SKLU1 are also shown in Bortezomib, Idarubicin, Tanespimycin, SN-38, and Gemcitabine.

For 13 among 17 small molecules, ACE2 expression levels at 6 dose points in A549 were available in the preprocessed dataset. The dose-response of ACE2 expression levels in each small molecule are shown in Figure 2. The top 6 small molecules with lower ACE2 levels in A549 in Table 1 (acetylcysteine, CD-437, NVP-BEZ235, amifostine, ingenol, and NVP-AUY922) decreased ACE2 expression almost dose-dependently within the ranges up to their optimal doses. These small molecules are expected to decrease ACE2 expression in A549, human adenocarcinoma alveolar basal epithelial cells.

### *In-vitro* Pipeline for Evaluation of Predicted Drug Repurposing Candidates That Reduce ACE2 Expression in Human Lung Epithelial Cells

#### Evaluation of Cytotoxicity Profile of Drug Repurposing Candidates in Normal Human Immortalized Bronchial Epithelial Cells

The first *in vitro* step in our drug repurposing validation pipeline was to evaluate the effect of the various predicted small molecules in impacting cellular health and viability. As listed in Table 1, these compounds have a wide range of mechanisms of action. Stringent characterization of the effect of a treating relevant cell type with these compounds was therefore performed.

The five lung epithelial cell lines utilized in the L1000 were tumor-derived lung epithelial cell lines. Evaluating the effectiveness of these predicted drug repurposing candidates in reducing ACE2 expression in COVID-19 infected patients, however, required analysis in a non-tumor lung epithelial cell setting. BEAS-2B is a normal human immortalized bronchial epithelial cell line, which has been extensively used to study cellular and molecular mechanisms involved in lung. This study therefore used BEAS-2B as a host cell model that could be infected by SARS-CoV-2.

**Figure 3:**
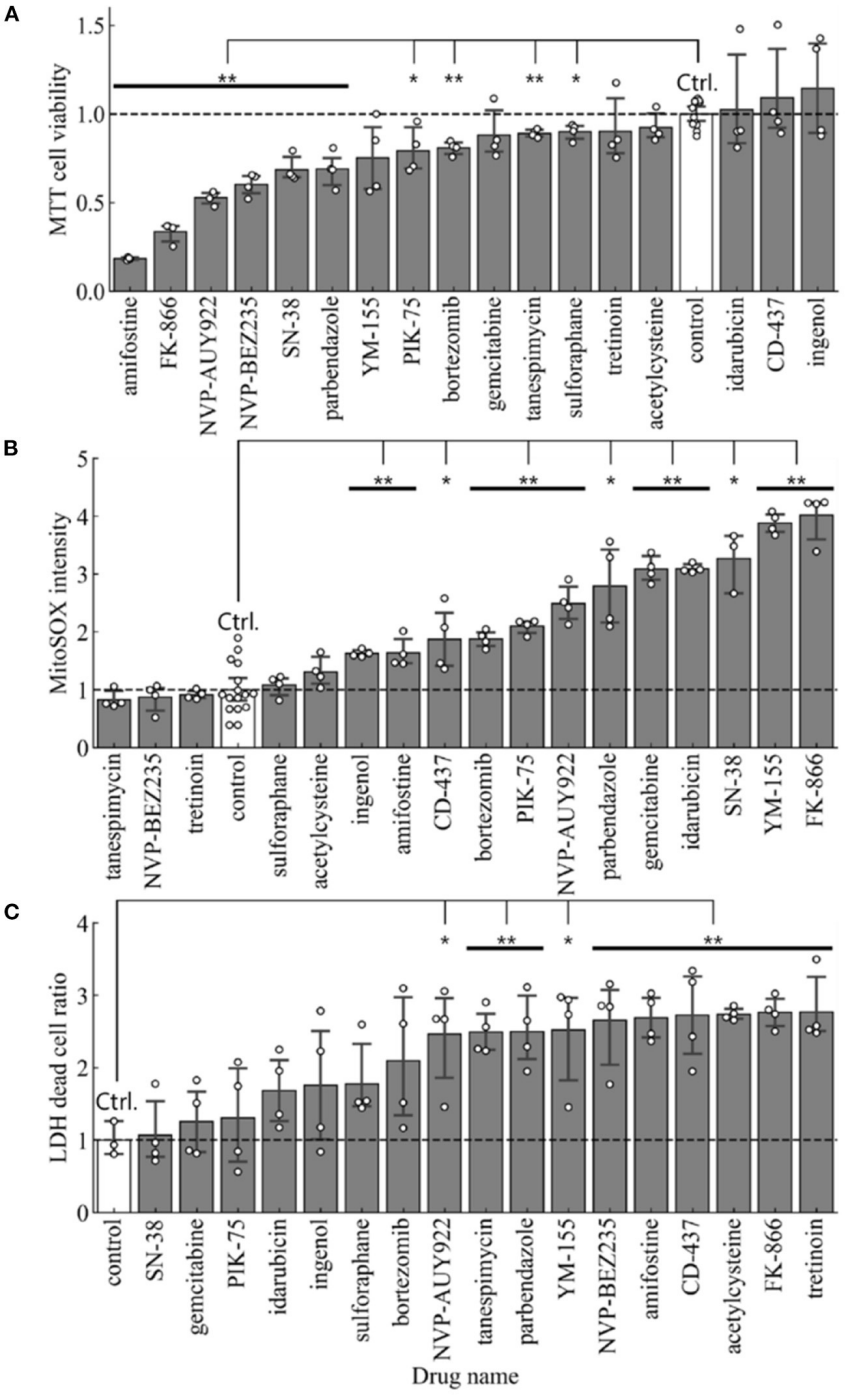
Cell toxicity assay in BEAS-2B treated with each of 17 small molecules and the control. **(A)** Cell viability measured by MTT assay (*n* = 4 in samples and *n* = 12 in control), **(B)** Mitochondrial Super Oxide production measured by MitoSOX assay (*n* = 4 in samples and *n* = 16 in control), and **(C)** Cytotoxicity measured by LDH assay (*n* = 4). Error bars mean 95% confidence interval. *p < 0.05, **p < 0.01.

For cell toxicity studies, the BEAS-2B cells were treated with indicated amounts of each drug repurposing candidate, and cell health was evaluated using multiple orthogonal measures. MTT assay measured the amount viable cells in each well by quantifying the amount of MTT converted to formazan crystals. The average absorbance in cells receiving vehicle were used to normalize the effect of each drug repurposing candidate. Therefore, a ratio <1.0 indicates treatments which exerted a negative effect on cell viability when compared to control cells. In this analysis, all compounds other than CD-437, Idarubicin and Ingenol had a ratio <1.0 (Figure 3A). Comparison of cell viability using Student's *t*-test (*p* < 0.05) showed that 10 out of 17 drug repurposing candidates had a statistically significant lower viability as indicated by MTT assay.

To further characterize whether mitochondrial stress was present upon treatment with these drug re-purposing candidates, we evaluated mitochondrial specific superoxide species production using MitoSOX. We used an HCI strategy to quantify the intensity of mitochondrial superoxide species and compared the average intensity of mitochondrial superoxide per cell in each treatment. A ratio >1.0, therefore, indicated higher levels of mitochondrial reactive oxygen species production upon treatment with the respective drug-repurposing candidates as described in Figure 3B. NVP-BEZ235, Tanespimycin and Tretinoin were the three compounds with ratio <1.0. All other compounds had elevated mitochondrial superoxide species when compared to control treatment. Comparison of MitoSOX intensity using Student's *t*-test (*p* < 0.05) showed that 12 out of 17 drug repurposing candidates had a statistically significant increase in mitochondrial stress as indicated by MitoSOX staining.

Finally, we used a third method to characterize the effect of these drug repurposing candidates in cell cytotoxicity using Lactate Dehydrogenase (LDH) assay. This assay measures the amount of LDH released out into the supernatant from dead cells with leaky plasma membrane. All readouts were normalized to wells where 100% of cells were lysed using a cell lysis buffer. Compared to this number, the average percentage of dead cells in each well was calculated. Again, percent cell death was normalized to control wells receiving vehicle treatment (Figure 3C). Therefore, a ratio >1.0 indicates elevated levels of cytotoxicity. All drug repurposing candidates showcased a ratio >1.0 indicated increased cell death upon treatment. Comparison of cell death using Student's *t*-test (*p* < 0.05) showed that 10 out of 17 drug repurposing candidates had a statistically significant increased cell death as indicated by LDH cytotoxicity assay.

#### Consensus Ranking of Cytotoxicity in Normal Human Lung Epithelial Cell Lines

To compile the three formats used to evaluate cytotoxicity in BEAS-2B cells treated with our drug repurposing candidates, we built a consensus ranking table comprising of MTT, MitoSOX and LDH cytotoxicity assays. Table 2 depicts the consensus ranking based on these three assays and ranks small molecules based on low cytotoxicity across all three formats. In this consensus ranking analysis, the following seven candidates, Ingenol, Sulforaphane, Tanespimycin, Idarubicin, CD-437, PIK-75, and Gemcitabine showed a consistently low cytotoxic profile at indicated doses in BEAS-2B lung epithelial cells. This identification of small molecules with favorable safety profile in target cell type is required for our drug repurposing pipeline in order to find suitable candidates for treatment where low cytotoxic side effects are crucial for applicability of identified small molecules.

**Table 2:** Consensus ranking comprising of MTT, MitoSOX, and LDH cytotoxicity assay.

**Drug name**	**Cyto toxicity ranking**			
	**MTT**	**MitoSOX**	**LDH**	**Ave**.
Control	4	4	1	3.0
Ingenol	1	7	6	4.7
Sulforaphane	7	5	7	6.3
Tanespimycin	8	1	10	6.3
Idarubicin	3	15	5	7.7
CD-437	2	9	15	8.7
PIK-75	11	11	4	8.7
Gemcitabine	9	14	3	8.7
Acetylcysteine	5	6	16	9.0
Tretinoin	6	3	18	9.0
Bortezomib	10	10	8	9.3
NVP-BEZ235	15	2	13	10.0
SN-38	14	16	2	10.7
NVP-AUY922	16	12	9	12.3
Parbendazole	13	13	11	12.3
Amifostine	18	8	14	13.3
YM-155	12	17	12	13.7
FK-866	17	18	17	17.3

*Each value means ranking of low toxicity (i.e., high number means high toxicity). Drugs are sorted based on the average ranking over 3 assays*.

#### Evaluation of ACE2 Surface Expression in Human Lung Epithelial Cells With Predicted Candidates for Drug Repurposing

**Figure 4:**
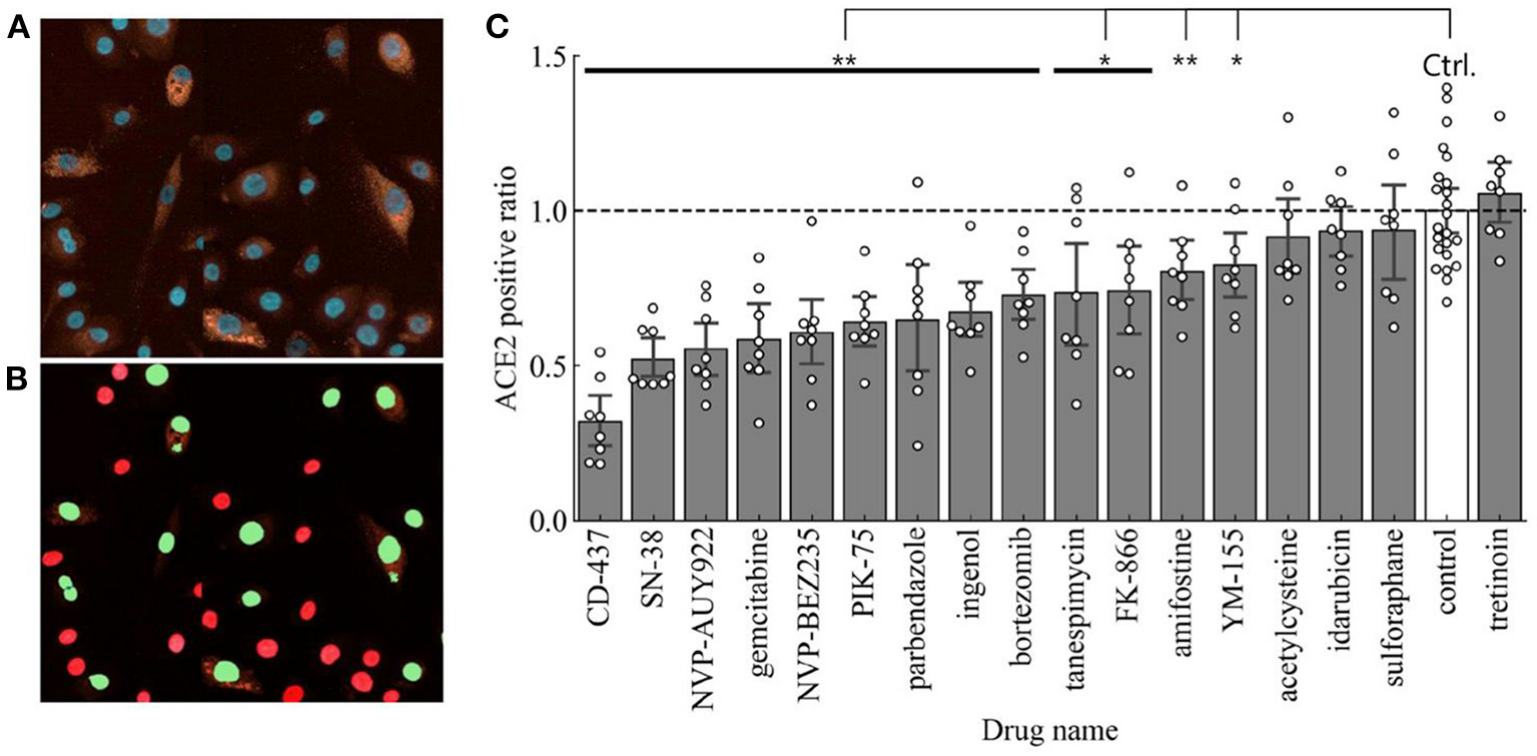
**(A)** Representative image showing ACE2 staining in BEAS-2B. ACE2-high expressed (ACE2 positive) cells are detected and then ACE2 positive ratio is calculated. **(B)** Representative cell scoring masks indicating ACE2 expressing cells (Green) and ACE2 negative cells (Red). **(C)** ACE2 positive ratios in each drug treatment compared to the control (*n* = 8 in samples and *n* = 23 in control). Error bars mean 95% confidence interval. *p < 0.05, **p < 0.01.

The next step in our drug repurposing pipeline was to determine the effect of these predicted drug repurposing candidates to reduce surface expression of ACE2. For this study, ACE2 surface expression serves as the most important determinant since we were evaluating the capacity of these small molecules to reduce the prevalence of surface receptors to which the SARS-CoV-2 spike protein can bind to and subsequently infect target cells. We thus used immuno-fluorescent staining to quantify surface expression of human ACE2 expression using HCI followed by a cell scoring system using red staining intensity for ACE2 and cell calling using Hoescht 33342 nuclear counterstain. A representative image for ACE2 staining in BEAS-2B is shown in Figure 4A. Thresholds for determining ACE2 expression were established using cells stained with isotype primary antibody followed by secondary antibody staining. Cell calling was performed using nuclear counter stain and establishing appropriate cell diameter to detect all cells within 4 different fields of view. Using these parameters, we observed cells that were high for ACE2 expression and others that expressed ACE2 at low levels (Figure 4B). Of note, even when looking with the normal immortalized cell line, we observed heterogeneity in ACE2 expression across cells. Using this categorization strategy, we will use the nomenclature of ACE2 high- or low-expressed cells to determine the effect of our various treatments in modifying ACE2 expression in a lung epithelial cell population. The number ratios of high-expressed cells in the total cell number were calculated as ACE2 positive ratios in each small molecule treatment and were normalized with the ACE2 positive ratio of the control (no treatment).

The ACE2 positive ratios after our candidate treatments are shown in Figure 4C. Using Student's *t*-test (*p* < 0.05) to compare to control treated BEAS-2B cells, 13 out of 17 treatments showed a statistically significant reduction in surface ACE2 expression. In this regard, the top 3 small molecules with the higher decreasing level of ACE2 positive ratio are CD-437, SN-38, and NVP-AUY922.

#### Integration of ACE2 Expression and Cell Viability Data to Identify Top Drug Repurposing Candidates

**Figure 5:**
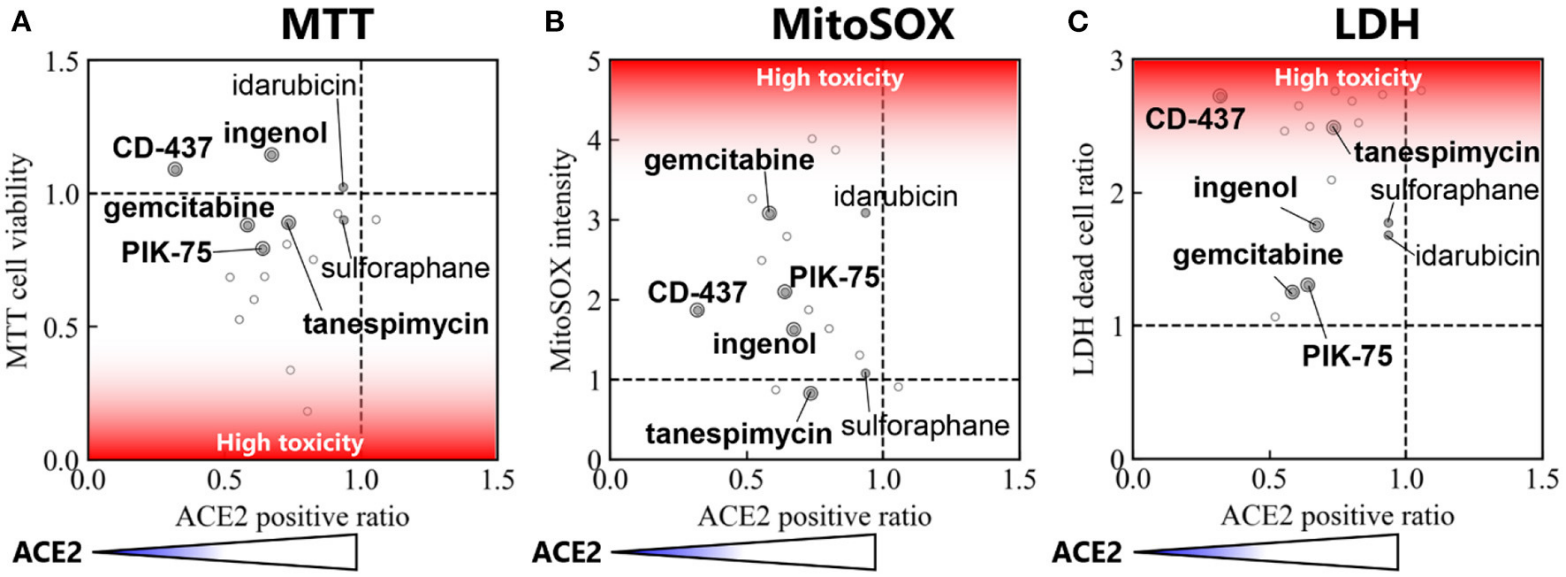
Correlation between ACE2 positive ratio vs. cell toxicity. Correlation between ACE2 positive ratio and **(A)** cell viability measured by MTT assay, **(B)** MitoSOX Assay, and **(C)** LDH assay. The locations of the top 7 small molecules with reduced cytotoxicity in Table 2 are labeled.

This pipeline follows the L1000 powered identification of small molecule candidates for drug repurposing by evaluating the effect of these compounds in affecting both cell health and surface ACE2 expression. Therefore, correlation analysis was performed in order to narrow down the candidates and identify small molecules that could be used to reduce ACE2 expression without inducing high levels of cell death specifically in our target cell type BEAS-2B (Figure 5). Three correlation matrices were constructed to compare the effect of each candidate in affecting cell health as well as the potency with which they reduced ACE2 expression. Using this matrix, Ingenol, CD-437, Tanespimycin, PIK-75, and Gemcitabine were five compounds that consistently showed low cytotoxicity and effective downregulation of surface ACE2 expression. This data suggests that these five candidates identified using our novel drug repurposing pipeline can be evaluated for large scale studies in preventing SARS-CoV-2 infection by targeting ACE2 expression levels.

#### Validation of Identified Top Candidates Combining Viability and ACE2 Expression

**Figure 6:**
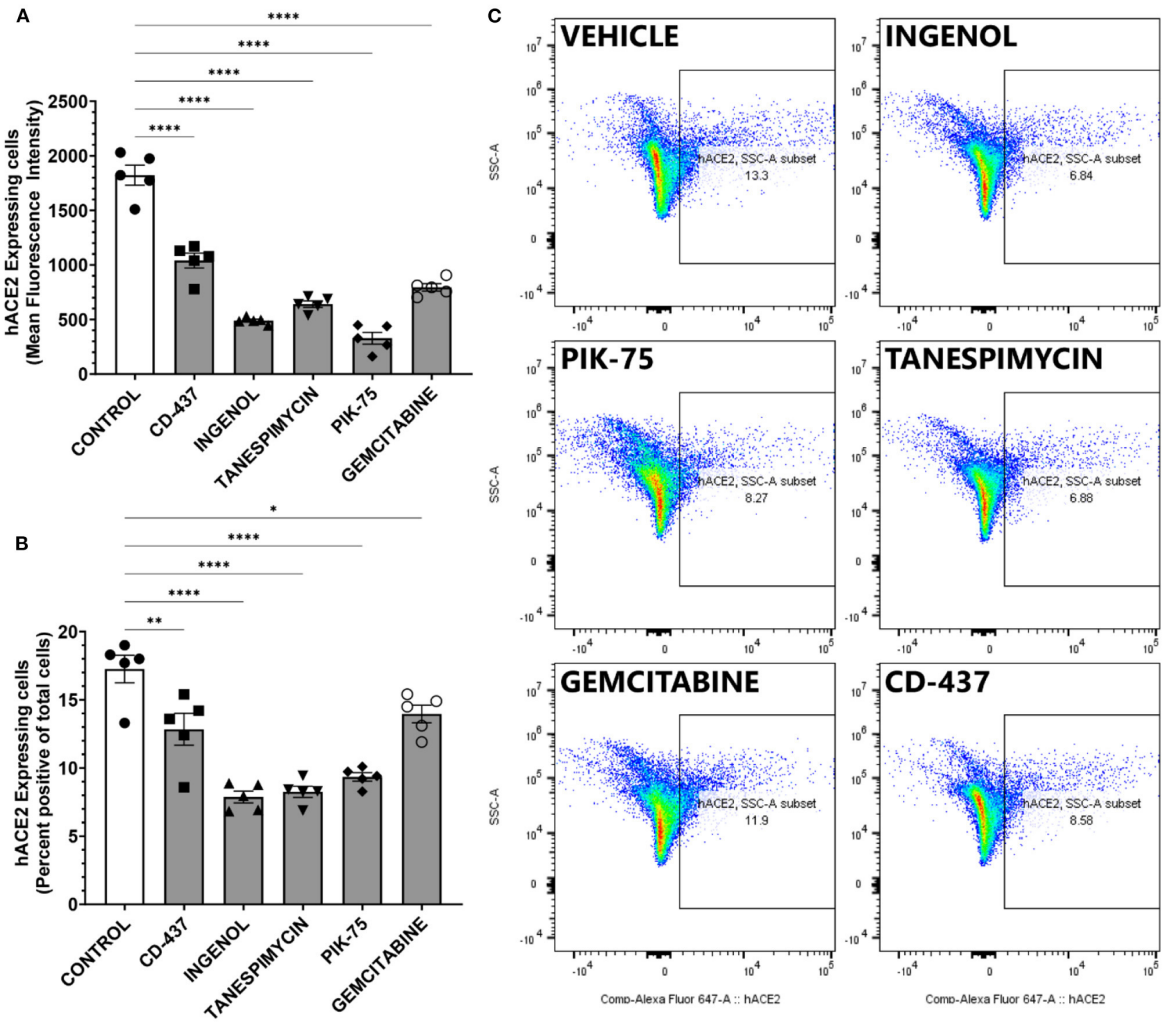
ACE2 expression measure by FACS in BEAS-2B treated with the 5 repurposing candidates. **(A)** mean ACE2 expression (*n* = 5). **(B)** ACE2 positive ratio. Error bar means standard deviation (*n* = 5). *p < 0.05, **p < 0.01, ****p < 0.0001. **(C)** Representative scatter plot showing percent positive ACE2 surface expression in BEAS-2B cells.

The top five candidates with low toxicity and potent decrease in ACE2 expression were further validated using an orthogonal platform, Fluorescence Assisted Cell Sorting. This platform allows for the quantification of surface ACE2 expression in viable, living cells. Furthermore, quantitative analysis can be performed in an unbiased fashion in a large number of cells. BEAS-2B cells were treated with indicated doses of drugs and then percent positive cells for surface ACE2 were determined using control cells treated with isotype antibody (Supplementary Figure 1). All five candidates produced statistically significant reductions in both the overall amount of surface ACE2 receptors and the mean fluorescent intensity of ACE2 staining in viable BEAS-2B cells (Figure 6A). Furthermore, the fraction of cells determined to be positive for ACE2 expression (Figures 6B,C) also showed statistically significant reductions with all five compounds identified using our pipeline. Taken together, our data suggest that intervention with these five small molecule compounds can decrease the number of viable cells susceptible to SARS-CoV-2 infection by altering surface ACE2 expression.

## Discussion

We identified 17 candidate small molecules that possibly decrease ACE2 expression by processing the L1000-based CMap dataset with focusing on the drug repurposing for COVID-19 (Table 1 and Figure 1). These candidates decreased ACE2 mRNA levels within 6 h in the lung epithelial cell lines in the preprocessed dataset (Table 1 and Figure 2). This suggests that the decrease in ACE2 mRNA levels likely led to reduced ACE2 surface expression in the target cells BEAS-2B derived from the same organ. Indeed, most candidates decreased ACE2 expression on the surface of BEAS-2B (Figure 4). These results indicate that our L1000-powered drug screening effectively identifies small molecules that modulate a single drug target. Further investigation, however, is required to address the mechanism of action for ACE2 suppression by these compounds as well as the effects on ACE2 expression in other cell types.

On the other hand, the identified small molecules were mostly drugs that have been developed for cancer treatment, therefore, their cytotoxic effects in BEAS-2B were evaluated using MTT, MitoSOX, and LDH assays. Over half candidates showed significant cytotoxicity or cell viability reduction in each assay (Figure 3). To identify small molecules that consistently show a low cytotoxic profile, we built a consensus ranking table (Table 2). The top 2 small molecules with the lowest toxicity were Ingenol and Sulforaphane. Ingenol is an FDA-approved drug for keratosis. Sulforaphane is a naturally occurring isothiocyanate found in cruciferous vegetables such as broccoli. These facts support the validity of this table.

Our goal was to obtain the drug repurposing candidates that could be used to reduce surface ACE2 expression without inducing high cell toxicity. Therefore, correlation analysis was performed to narrow down the candidates (Figure 5). In this correlation matrix, Ingenol, CD-437, Tanespimycin, PIK-75, and Gemcitabine consistently showed low cytotoxicity and effective downregulation of surface ACE2 expression. These were five compounds, out of the top 7 with low toxicity in Table 2, that show significant ACE2 reduction in Figure 4. Moreover, additional validation experiments demonstrated that all these five candidates decreased the surface ACE2 expression in living BEAS-2B (Figure 6). These results suggest that these five candidates can be evaluated for large scale studies in preventing SARS-CoV-2 infection by targeting ACE2 expression levels.

Our proposed pipeline consists of L1000-powered drug screening and the further narrowing of the drug repurposing candidates based on their cytotoxic effect. As described above, the L1000 screening can be applied to a single target, allowing us to apply this method to target-based drug discovery which is a gold standard strategy. The small molecules identified by this method are different from those by conventional screenings like HTS because our approach focuses on the target gene mRNA level instead of the target protein. In addition, conventional L1000-CMap approaches have no applicability to target-based drug discovery because it requires a gene set rather than a single gene. Furthermore, our pipeline can be adapted to a wide range of cell types including endothelial cells and monocytes. This is of interest since viruses including HIV and SARS-CoV-2 have been shown to specifically induce pulmonary endothelial cell activation (16, 17). Our cell types of interest in the present study were epithelial cells. We thus used the identified compounds in this cell type to perform a series of assays to evaluate cell health and stress. COVID-19, however, affects other cell types. Application of our pipeline to other cell types could include various additional functional assays depending on the cell type of interest, for example, angiogenesis assays in endothelial cells, or measuring pro-inflammatory chemokine secretion in monocytes. Thus, our pipeline provides a novel screening method that is different from both HTS and conventional L1000-CMap approaches, contributing to the repurposing of FDA approved drugs to combat rapidly emerging diseases as well as other diseases like vascular calcification that conventional approaches have yet found the therapeutic options.

## Data Availability Statement

Publicly available datasets were analyzed in this study. This data can be found here: https://www.ncbi.nlm.nih.gov/geo/query/acc.cgi?acc=GSE92742; https://www.ncbi.nlm.nih.gov/geo/query/acc.cgi?acc=GSE70138.

## Author Contributions

TA and SC: conception and design, collection of data, data analysis and interpretation, manuscript writing, and final approval of the manuscript. JD: conception and design and final approval of the manuscript. MW: collection of data, data analysis and interpretation, and final approval of the manuscript. EA: financial support, administrative support, and final approval of the manuscript. MA: conception and design, financial support, administrative support, data interpretation, manuscript editing, and final approval of the manuscript. All authors contributed to the article and approved the submitted version.

## Funding

The authors declare that this study received funding from Kowa Company, Ltd, Nagoya, Japan (research grant A11014 to MA). The funder was not involved in the study design, collection, analysis, interpretation of data, the writing of this article or the decision to submit it for publication.

## Conflict of Interest

TA is an employee of Kowa Company, Ltd and was a visiting scientist at Brigham and Women's Hospital when experiments demonstrated in this study were performed. The remaining authors declare that the research was conducted in the absence of any commercial or financial relationships that could be construed as a potential conflict of interest.

## Publisher's Note

All claims expressed in this article are solely those of the authors and do not necessarily represent those of their affiliated organizations, or those of the publisher, the editors and the reviewers. Any product that may be evaluated in this article, or claim that may be made by its manufacturer, is not guaranteed or endorsed by the publisher.

## References

[1] Brandão SCS Ramos J de OX Dompieri LT Godoi E. T. A. M.. Is Toll-like receptor 4 involved in the severity of COVID-19 pathology in patients with cardiometabolic comorbidities? Cytok Growth Factor Rev. (2021) 58:102–10. 10.1016/j.cytogfr.2020.09.002 32988728 PMC7505161

[2] Jha PK Vijay A Halu A Uchida S Aikawa M. Gene expression profiling reveals the shared and distinct transcriptional signatures in human lung epithelial cells infected with SARS-CoV-2, MERS-CoV, or SARS-CoV: potential implications in cardiovascular complications of COVID-19. Front Cardiovasc Med. (2021) 7:1–15. 10.3389/fcvm.2020.623012 33521069 PMC7844200

[3] Lu H Chelvanambi S Poirier C Saliba J March KL Clauss M. EMAPII monoclonal antibody ameliorates influenza A virus-induced lung injury. Mol Ther. (2018) 26:2060–9. 10.1016/j.ymthe.2018.05.017 29910176 PMC6094359

[4] Chelvanambi S Gupta SK Chen X Ellis BW Maier BF Colbert TM. HIV-Nef protein transfer to endothelial cells requires Rac1 activation and leads to endothelial dysfunction: implications for statin treatment in HIV patients. Circ Res. (2019) 125:805–20. 10.1161/CIRCRESAHA.119.315082 31451038 PMC7009312

[5] Nishiga M Wang DW Han Y Lewis DB Wu JC. COVID-19 and cardiovascular disease: from basic mechanisms to clinical perspectives. Nat Rev Cardiol. (2020) 17:543–58. 10.1038/s41569-020-0413-9 32690910 PMC7370876

[6] Rabi FA Al Zoubi MS Al-Nasser AD Kasasbeh GA Salameh DM. Sars-cov-2 and coronavirus disease 2019: what we know so far. Pathogens. (2020) 9:231. 10.3390/pathogens9030231 32245083 PMC7157541

[7] Gil C Ginex T Maestro I Nozal V Barrado-Gil L Cuesta-Geijo MÁ. COVID-19: drug targets and potential treatments. J Med Chem. (2020) 63:12359–86. 10.1021/acs.jmedchem.0c00606 32511912

[8] Kifle ZD Ayele AG Enyew EF. Drug repurposing approach, potential drugs, and novel drug targets for COVID-19 treatment. J Environ Public Health. (2021) 2021:6631721. 10.1155/2021/6631721 33953756 PMC8063850

[9] Swinney DC Anthony J. How were new medicines discovered? Nat Rev Drug Discov. (2011) 10:507–19. 10.1038/nrd3480 21701501

[10] Lamb J Crawford ED Peck D Modell JW Blat IC Wrobel MJ. The Connectivity Map: using gene-expression signatures to connect small molecules, genes, and disease. Science. (2006) 313:1929–35. 10.1126/science.1132939 17008526

[11] Subramanian A Narayan R Corsello SM Peck DD Natoli TE Lu X. A next generation connectivity map: L1000 platform and the first 1,000,000 profiles. Cell. (2017) 171:1437–52.e17. 10.1016/j.cell.2017.10.049 29195078 PMC5990023

[12] Duan Q Reid SP Clark NR Wang Z Fernandez NF Rouillard AD. L1000CDS2: LINCS L1000 characteristic direction signatures search engine. Npj Syst Biol Appl. (2016) 2:1–12. 10.1038/npjsba.2016.15 28413689 PMC5389891

[13] Musa A Ghoraie LS Zhang SD Glazko G Yli-Harja O Dehmer M. A review of connectivity map and computational approaches in pharmacogenomics. Brief Bioinform. (2018) 19:506–23. 10.1093/bib/bbw112 28069634 PMC5952941

[14] Swinney DC. Phenotypic vs. Target-based drug discovery for first-in-class medicines. Clin Pharmacol Ther. (2013) 93:299–301. 10.1038/clpt.2012.236 23511784

[15] Corsello SM Bittker JA Liu Z Gould J McCarren P Hirschman JE. The drug repurposing hub: a next-generation drug library and information resource. Nat Med. (2017) 23:405–8. 10.1038/nm.4306 28388612 PMC5568558

[16] Chelvanambi S Bogatcheva NV Bednorz M Agarwal S Maier B Alves NJ. HIV-Nef protein persists in the lungs of aviremic patients with HIV and induces endothelial cell death. Am J Respir Cell Mol Biol. (2019) 60:357–66. 10.1165/rcmb.2018-0089OC 30321057 PMC6397978

[17] Clauss M Chelvanambi S Cook C Elmergawy R Dhillon N. Viral bad news sent by evail. Viruses. (2021) 13:1–13. 10.3390/v13061168 34207152 PMC8234235

