# Supplementary material for: In silico Drug Screening Approach Using L1000-Based Connectivity Map and Its Application to COVID-19
Source: Front Cardiovasc Med. 2022 Mar 24;9:842641. doi: 10.3389/fcvm.2022.842641 (PMC8989014; doi:10.3389/fcvm.2022.842641)
Supplement: Supplementary file 1 [file Data_Sheet_1.docx]

Supplementary TABLE S1 | Look-up table to convert doses to the representative values.

| Range (μM) | Representative Value (μM) |
| --- | --- |
| 0 - 0.000178 | 0.0001 |
| 0.000178 - 0.000562 | 0.000316 |
| 0.000562 - 0.00178 | 0.001 |
| 0.00178 - 0.00562 | 0.00316 |
| 0.00562 - 0.0178 | 0.01 |
| 0.0178 - 0.0562 | 0.0316 |
| 0.0562 - 0.178 | 0.1 |
| 0.178 - 0.562 | 0.3162 |
| 0.562 - 1.78 | 1 |
| 1.78 - 5.62 | 3.16 |
| 5.62 - 17.8 | 10 |
| 17.8 - 56.2 | 31.6 |
| 56.2 - 178 | 100 |


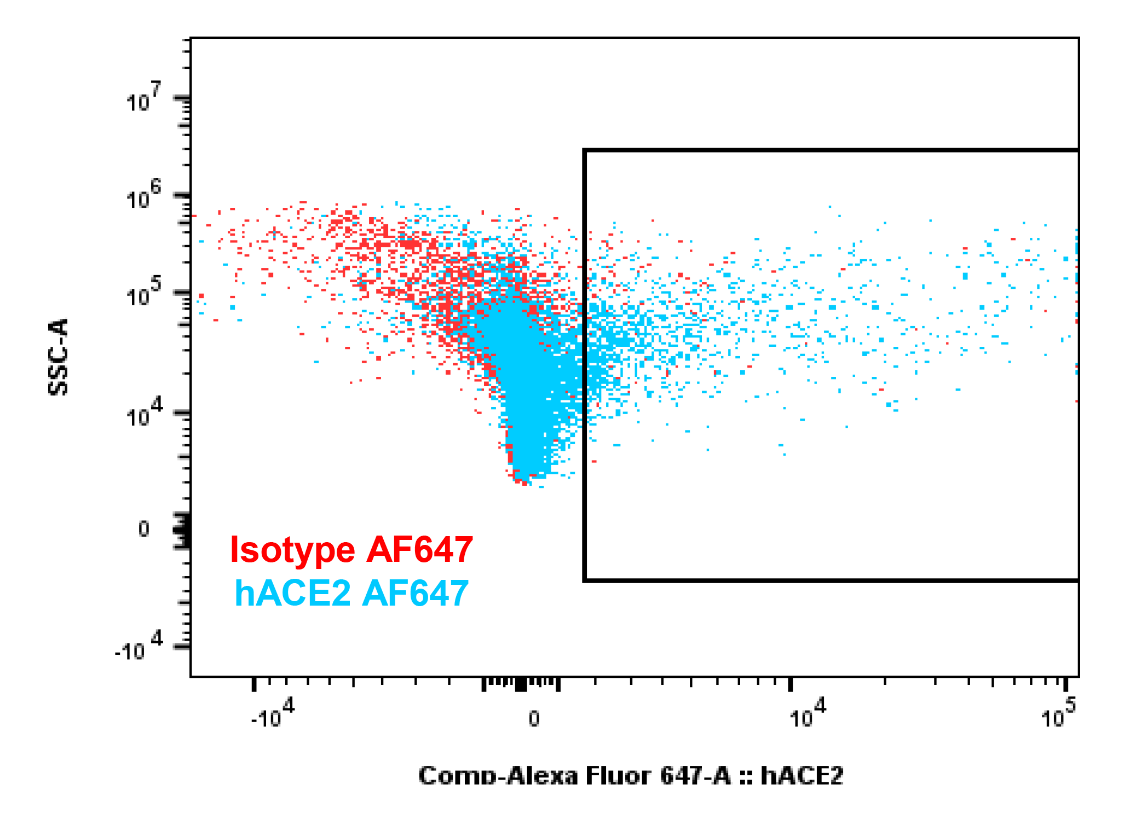


**Supplementary FIGURE S1** | Representative scatter plot showing gating strategy for ACE2 positivity using isotype AF647 and hACE2 AF647 stained BEAS-2B cells.
